# Scrofula; Its Nature, Its Prevalence, and the Principles of Treatment

**Published:** 1846-11

**Authors:** 


Art. VIII.—*Scrofula; its Nature, its Prevalence, and the Principles of Treatment.* By Benjamin Philips, F.R.S., &c. Philadelphia: Lea & Blanchard. 8vo. pp. 350.

An analytical review of this work would have appeared in the present number of the Journal, but for the elaborate prize essay of Dr. Sutton on that subject, which has been admitted to the exclusion of nearly everything else. If not in our next number, at an early day, the notice will appear, and, meantime, we commend the work to the profession as the best treatise that has ever been written on Scrofula.

